# Recent Insights about the Role of Gels in Organic Photonics and Electronics

**DOI:** 10.3390/gels9110875

**Published:** 2023-11-04

**Authors:** Josué M. Galindo, Carlos Tardío, Basanta Saikia, Stijn Van Cleuvenbergen, Iván Torres-Moya

**Affiliations:** 1 Department of Chemistry, RCSI University of Medicine and Health Sciences, 123 St. Stephen’s Green, D02YN77 Dublin, Ireland; josuemunozgalindo@rcsi.ie; 2 Department of Inorganic, Organic Chemistry and Biochemistry, Faculty of Chemical Science and Technologies, University of Castilla-La Mancha-IRICA, 13071 Ciudad Real, Spain; carlos.tardio@uclm.es; 3 Department of Chemistry, Molecular Imaging and Photonics, KULAK—KU Leuven, E. Sabbelaan 53, 8500 Kortrijk, Belgium; basanta.saikia@kuleuvern.be (B.S.); stijn.vancleuvenbergen@kuleuven.be (S.V.C.); 4 Department of Organic Chemistry, Faculty of Chemical Sciences, Campus of Espinardo, University of Murcia, 30010 Murcia, Spain

**Keywords:** gels, organic field-effect transistors, solar cells, organic light-emitting diodes, optical waveguides

## Abstract

This review article provides an in-depth exploration of the role of gels in the fields of organic electronics and photonics, focusing on their unique properties and applications. Despite their remarkable potential, gel-based innovations remain relatively uncharted in these domains. This brief review aims to bridge the knowledge gap by shedding light on the diverse roles that gels can fulfil in the enhancement of organic electronic and photonic devices. From flexible electronics to light-emitting materials, we delve into specific examples of gel applications, highlighting their versatility and promising outcomes. This work serves as an indispensable resource for researchers interested in harnessing the transformative power of gels within these cutting-edge fields. The objective of this review is to raise awareness about the overlooked research potential of gels in optoelectronic materials, which have somewhat diminished in recent years.

## 1. Introduction

Gels can be defined as semisolid, crosslinked systems containing condensed solid particles interpenetrated by a liquid phase that can be water or organic solvents. The first reports about supramolecular gels date back to the 1930s [1], in which a gel-like substance was obtained from an organic molecule, representing a major breakthrough in recent supramolecular chemistry. However, it is important to note that the concept of “gel” had already been introduced to the scientific community before the 1930s. We must point out that, in 1861, Thomas Graham gave the first description of the term “gels” [2] and after that, Dorothy Jordon Lloyd actualized the previous definition in 1926 [1]. The combination of both of them can allow us to obtain an appropriate definition of “gels”. Despite their discovery in 1930, gels did not receive considerable attention from the scientific community, with only a few studies focusing on their use as thickeners and lubricants in subsequent years. However, their significant resurgence in research and development emerged during the 1970s and 1980s, with a boom in the 2000s leading to a multitude of studies and diverse applications of them. 

In general, a substance is a gel if (1) it has a continuous microscopic structure with macroscopic dimensions which is permanent on the time scale of analytical experiments and (2) is solid-like in its rheological behavior, despite being mostly liquid. Organogels and hydrogels, two distinct classes of gels, have wide-ranging applications. Organogels involve semisolid systems entrapping organic solvents within a self-assembled molecular network, while hydrogels, water-based gels, are renowned for their water retention capabilities and biocompatibility. 

The potential of gels is well known and evidenced by the large amount of research dedicated in this field in recent years. Biological applications of gels are paramount these days in the fields of medicine, pharmaceuticals, and biotechnology. There are numerous examples of gel applications found, for example, in sensors [3,4,5], actuators [6,7,8], tissue engineering [9,10], drug-delivery systems [11,12,13], and drug crystallization [14,15,16]. In addition to well-developed applications, other emerging ones can be found such as: 3D/4D printing [17,18], food and related applications [19,20,21], energy storage [22,23], agriculture applications [24,25], or even applications in the cosmetic industry [26,27,28]. 

Despite the undeniable advancement of gels in the field of biomedicine and related fields, applications in other fields like photonics and electronics have not yet been sufficiently exploited. Considering this, gel materials present a compelling opportunity to harness the potential application in the last-mentioned fields.

**Figure 1:**
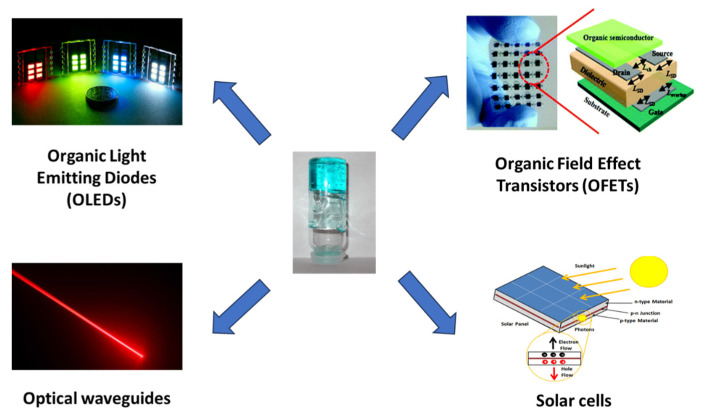
Different fields of application of gels in photonics and electronics.

In this review article, we aim to reflect on the potential and the possibilities that gels can offer in the fields of photonics and electronics. In this regard, we will briefly discuss the advances that the gels have brought in organic field-effect transistors (OFETs), solar cells, organic light-emitting diodes (OLEDs), and optical waveguides (Figure 1).

## 2. Gels in Organic Field-Effect Transistors (OFETs)

In a current technological world, organic field-effect transistors (OFETs) have captured the attention of scientists, engineers, and the industry due to their uniqueness and versatile application in organic materials-based electronics. A standard OFET comprises key components: the gate electrode in which the voltage is applied, the source and drain electrodes that facilitate charge transport, and the dielectric layer that separates the gate electrode from the active layer, primarily composed of an organic semiconductor, crucial for current modulation. In contrast to conventional inorganic transistors (FETs) that rely on inorganic materials, OFETs harness the unique properties of organic materials to control the flow of electric current in a channel, enabling a wide range of innovative electronic applications. 

The integration of gels in OFETs offers several great advantages. Primarily, gels facilitate low-temperature processing, promoting energy efficiency and compatibility with various substrates. Secondly, their cost-effectiveness, often utilizing organic compounds, reduces the overall manufacturing expenses of OFETs. Thirdly, gel-based materials tend to yield improved film uniformity and reduced defects, enhancing device performance and reliability. Lastly, gels provide supramolecular structures that facilitate the self-assembly of organic molecules, resulting in well-ordered, high-quality active layers within OFETs. These advantages collectively highlight the promising role of gels in advancing the field of organic electronics.

Generally, the deposition of the organic active layer involves the self-assembly of the organic molecule in film to achieve the final device. Considering this premise, gels can be attractive supramolecular structures for OFETs in the self-assembly process [29]. This allows for production of OFETs in a more cost-effective and simple manner compared to inorganic compounds. Furthermore, it is essential that the self-assembly can occur at room temperature, making it a more efficient process in comparison with the classical and well-developed, highly sophisticated processes for the deposition of the active layer such us sublimation or vapor deposition, among others.

Therefore, a most important advantage that supports the use of gels in OFETs is the potential for molecular organization in the organic-solvent-assisted self-assembly during device fabrication. This leads to a significant enhancement in hole and electron mobility due to the strong π-orbitals overlap within the supramolecular aggregates formed through organogelation [30]. 

**Figure 2:**
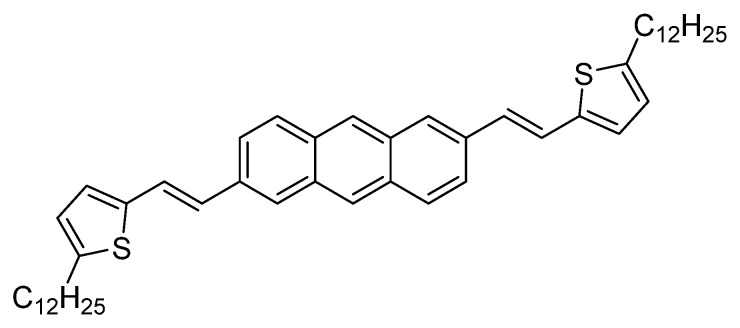
Molecular structure of dodecyl-substituted 2,6-bis(2-thienylvinyl)anthracene used to build gels to fabricate OFETs [31].

Lee and co-workers were the first to employ a gel-based system to the fabrication of OFETs [31]. They employed dodecyl-substituted 2,6-bis(2-thienylvinyl)anthracene (Figure 2) to build an OFET with an active layer constructed by the gelation of this compound arising from robust π–π stacking of the aromatic moiety and the presence of alkyl chains that favor van der Waals interactions. The hole mobilities were surprisingly high, up to 8.7 cm^2^V^−1^s^−1^.

These authors opened the door to the use of gels in OFETs and promoted other authors to continue researching in this field. However, the mobilities are not so high as in this first example, corroborating the need for deeper research in this field. 

**Figure 3:**
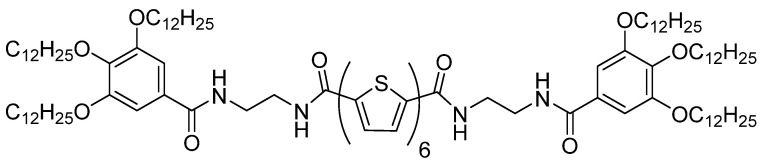
Molecular structure of the sextiophene derivative employed in this study [32].

For example, the gelation of a sexthiophene derivative described by Tsai and co-workers (Figure 3) allowed them to obtain active 1D nanofibers, useful for a semiconducting gel in OFETs [32]. In non-polar solvents, the molecule forms gels because of H-bonding, π-stacking, and van der Waals interactions. The OFET performance measurements revealed that the gel fibers exhibited hole mobility (3.46 × 10^−6^ cm^2^V^−1^s^−1^) that was higher than that of the isolated organic material without gelation (1.79 × 10^−7^ cm^2^V^−1^s^−1^). This highlights the ability of gels to enhance OFET performance even though these mobilities are relatively low.

**Figure 4:**
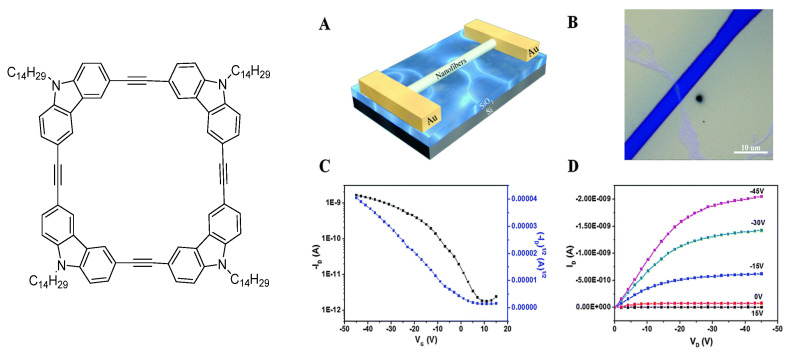
Molecular structure of the cyclic compound used for gelations to achieve an active layer in OFETs. (**A**) Schematic diagram of the device structure. (**B**) Optical image of the device based on single-bundle nanofibers. (**C**) Transfer and (**D**) output characteristics of the representative single-bundle nanofiber device based on the gelator molecule [33].

Hong and co-workers developed another cyclic π-gelator (Figure 4). Gelation of these molecules in methylcyclohexane allowed them to obtain well-organized fibers, which were employed for OFET fabrication. This approach yielded a remarkable hole mobility of 3.6 × 10^−3^ cm^2^V^−1^s^−1^, a substantial improvement compared to the significantly lower hole mobility of 6.7 × 10^−5^ cm^2^V^−1^s^−1^ in the non-assembled derivative (two magnitude orders) [33]. These devices displayed p-type semiconductor behavior with hole transport dominance in both dark and illuminated ambient conditions.

In these previous cases, the effect of the gel was presented in the active layer instead of the classical organic compound without gel self-assembly. However, gel can also improve the efficiency of the OFET when applied in the dielectric layer, generally comprising SiO_2_ or a polymer. For example, Kösemen demonstrates that using gel-based dielectric materials and molecular doping is a viable approach to enhance the performance of OFET devices [34]. To assess performance enhancement, PMMA and Poly(3-hexylthiophene-2,5-diyl) P3HT material systems were used as a reference. Propylene carbonate (PC) was introduced into PMMA to form the gel for use as a gate dielectric. The mobility increases from 5.72 × 10^−3^ to 0.26 cm^2^V^−1^s^−1^ and operation voltage decreases from −60 to −0.8 with the gel dielectric, corroborating the great improvement in the presence of the gel in the dielectric layer.

To the best of our knowledge, while significant progress has been made in developing p-type semiconductor gels, as previously described, there have been no reported instances in scientific literature of gels exhibiting n-type semiconductor behavior. Consequently, achieving ambipolar semiconductors through gels represents both an intriguing challenge and a promising avenue for future research in the field of electronic devices. Furthermore, research aimed at achieving ambipolar semiconductors in the form of gels is important, given the demonstrated potential of these materials in electronic applications. This research direction holds the promise of unlocking new opportunities in the design and fabrication of flexible and efficient electronic devices based on gels, taking advantage of the capacity of self-assembly in the film monolayer of these structures in the device.

## 3. Gels in Solar Cells

The last few decades have witnessed remarkable transformation in the area of organic electronics, with a focus on designing novel organic materials and their application in the manufacture of optoelectronic devices. This is closely linked to the development of new photovoltaic technologies, including organic solar cells (OSCs). These devices have aroused great interest within the scientific community and the industry due to their unique technical features such as lightness, flexibility, and reduced manufacturing costs, as well as the possibility of considerably lowering manufacturing costs by adapting their production using solution processing techniques.

In the recent years, there has been an important evolution in the field of solar cells from the use of fullerene acceptors [35,36] to the non-fullerene acceptors that occupy a large part of the current research in this field [37,38]. However, it is crucial to consider that the uses of these kind involves complex synthesis that hinders the efficiency of the whole process. For this reason, an innovative and interesting alternative could imply the further study of gels in this kind of devices.

In this sense, despite the fact that it is still unexplored, we can find some examples of the employment of gels in solar cells that opens the door for future modifications in order to increase their use in this field.

The most prominent example is the use of gel polymer electrolytes (GPEs) in dye-sensitized solar cells (DSSC). GPEs are usually manufactured capturing liquid electrolytes which contain organic solvents and inorganic salts. Among these, ethylene carbonate (EC), propylene carbonate (PC), sodium iodide (NaI), acrylonitrile (ACN), lithium iodide (LiI), and potassium iodide (KI) are the most commonly employed [39]. 

In GPE systems, the value of short-circuit density (J_sc_) usually decreases because of gelation. However, the open-circuit voltage (V_oc_) is improved and increased because of the suppression of a dark current thanks to polymer chains covering the TiO_2_ electrode’s surface [40]. Other parameters like power conversion efficiency (PCE) or fill factor (FF) also can be improved. In addition, the main advantages that promote the employment of GPEs include their low vapor pressure, superior wetting properties, and enhanced filling between the nanostructured electrode and counter-electrode. Additionally, GPEs exhibit higher ionic conductivity compared to conventional polymer electrolytes and greater thermal stability [39]. These attributes collectively contribute to the heightened stability of DSSCs.

**Figure 5:**
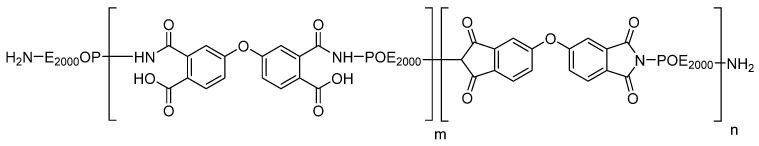
Poly(oxyethylene)-amideimide (POE-PAI), the molecule designed for the integration of PGE in DSSC [41].

An early and noteworthy instance illustrating the utilization of GPEs in DSSCs was documented in the research conducted by Jiang-Jen Lin and colleagues [41]. In this case, the GPE was formulated through the polymerization of poly(oxyethylene)-segmented diamine and 4,40-oxydiphthalic anhydride (Figure 5). A later-stage curing process was applied to achieve amide-imide crosslinked gels, ultimately yielding an elastomeric copolymer referred to as POE-PAI. This elastomer served as the framework for a PGE within the DSSC, leading to a remarkably high photovoltaic efficiency. The specially designed PGE, comprising 76.8 wt% of the liquid electrolyte, exhibited an impressive PCE of 9.48%, featuring a J_sc_ of 19.50 mA cm^2^, a V_oc_ of 0.76 V, and a FF of 0.64. The exceptional performance of the DSSC in gel-state, surpassing the DSSC with liquid electrolyte (8.84%), can be primarily attributed to the effective suppression of back electron transfer within the PGE.

**Figure 6:**
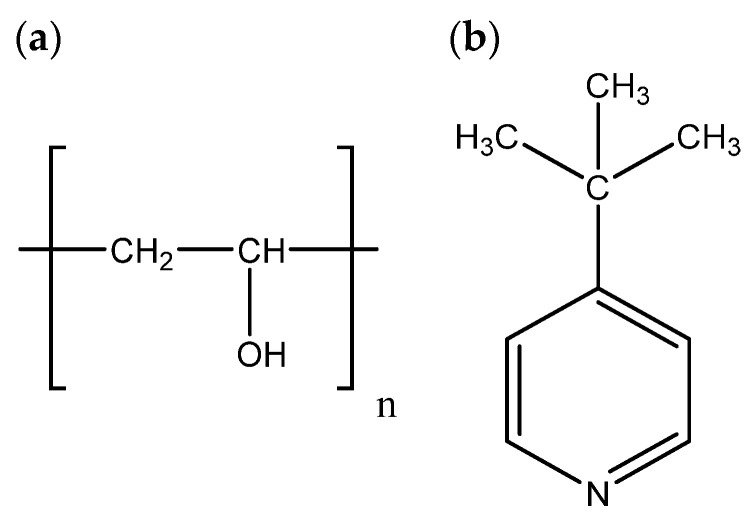
(**a**) Polyvinyl alcohol, (**b**) 4-tert-butylyridine [42].

Recently, another interesting example has been demonstrated by A. K. Arof and co-workers [42]. In this work, GPEs based on polyvinyl alcohol (Figure 6a) consisting of iodide/triiodide ions have been employed in DSSCs, investigating also the effect of 4-tert-butylpyridine (TBP) (Figure 6b) on the GPE and DSSC. The study of (J–V) graph characteristics of DSSCs reflected that the DSSC fabricated with TBP showed the highest Jsc (2.80 ± 0.30) mA/cm^2^ and PCE of 0.62%.

In addition, Jennings et al. present a dye-sensitized solar cell utilizing a natural agarose gel matrix, incorporating the photosystem I (PSI) protein complex to enhance device efficiency. Utilizing an agarose hydrogel facilitates redox reactions akin to those in a liquid device, all while simplifying the construction of a two-electrode apparatus [43].

**Figure 7:**
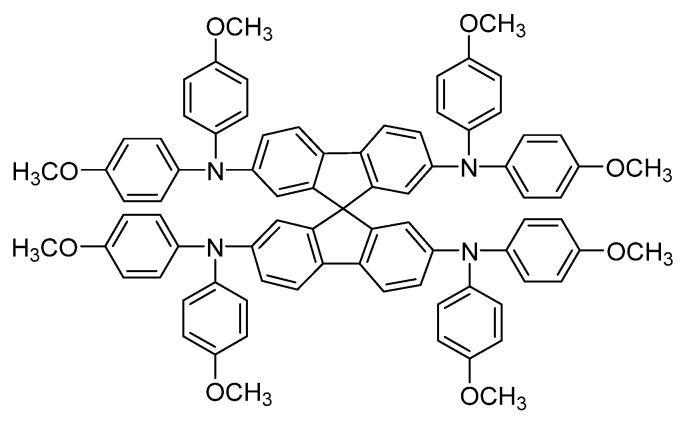
Chemical structure of Spiro-OMeTAD, a classical HTL in perovskite solar cells [44].

Although the most common application of gels in solar cells is as PGEs, as has been described, they can be also used, for example, in perovskite solar cells to improve their efficiency. It is well-known that, in perovskite solar cells, a HTL (hole transport layer) is commonly used to provide high conductivity, good moisture/oxygen barrier ability, and adequate passivation capability in order to improve the photovoltaic efficiency and the thermal stability of the solar cell [44]. Spiro-OMeTAD (Figure 7) is one of the most classical HTLs, but sometimes it is doped with a lithium compound (LiTFSI) to improve its behavior. However, the lithium salt dopant often induces crystallization and has a negative impact on the performance and lifetime of the device because of its hygroscopic character.

**Figure 8:**
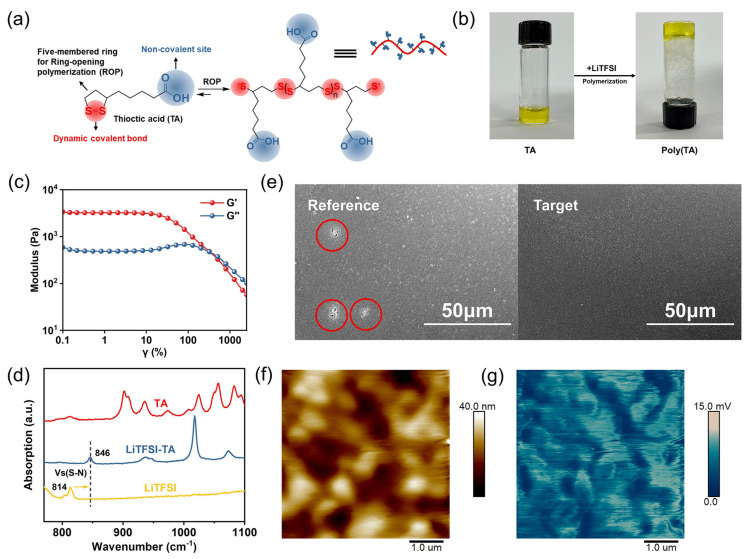
(**a**) Schematic representation of crosslinking polymerization of TA. (**b**) Pictures of the polymerization of TA. (**c**) Storage modulus (G′) and loss modulus (G″) for poly(TA) gels on strain sweep. (**d**) FTIR spectra of TA (red), mixture of LiTFSI and TA (blue), LiTFSI (yellow). (**e**) Scanning electron microscopy (SEM) images of spiro-OMeTAD and spiro-OMeTAD doped with TA films. (**f**) AFM images of Target film and (**g**) corresponding Nano-FTIR images [44].

To address this issue, this work provides a method of creating a gel via mixing a natural small-molecule additive (thioctic acid, TA) with spiro-OMeTAD. As a result, gelation effectively improves the compactness of the resultant HTL and prevents moisture and oxygen infiltration. Moreover, the gelation of the HTL improves, as does the operational robustness of the devices in the atmospheric environment. In addition, TA passivates the perovskite defects and facilitates the charge transfer from the perovskite layer to the HTL. As a consequence, the optimized PSCs based on the gelated HTL exhibit an improved PCE (22.52%) with excellent device stability (Figure 8).

Although there are few examples of gels in solar cells, the research should continue in the field of PGE to solve the limitations of the HTL in perovskite solar cells. The primary focus in this research field lies on the design of non-fullerene acceptors combining different acceptor and donor groups in different architectures, which inevitably leads to long and complicated synthesis. Hence, it would be worth leveraging the unique properties of gels to enhance the efficiency of solar cells. Easier to synthesize molecules with bisimide groups, which are involved in hydrogen bonding, facilitate gel formation or molecules with long alkyl chains can be used to form PGE and to dope the HTL, thereby increasing the efficiency of perovskite solar cells.

## 4. Gels in Organic Light-Emitting Diodes (OLEDs)

Organic light-emitting diodes, commonly known as OLEDs, represent solid-state devices with an integrated structure. They typically comprise a sequence of organic thin films enclosed between two conductive thin-film electrodes. When an electric current is applied to an OLED guided by an electric field, charge carriers, including holes and electrons, migrate from the electrodes into the organic thin films until they merge in the emissive area, forming excitons. Once these excitons, or heightened energy states, are established, they transition to a lower energy level by emitting light (known as electroluminescence) and/or undesired heat.

The fundamental configuration of an OLED cell involves a layering of thin organic materials placed between a conductive anode and a conductive cathode [45].

Its operation is more or less like the previously described OFETs, and the active layer is commonly constituted of an organic molecule or polymer [46,47,48,49]. However, gels are not usually used in these kind of devices (to a lesser extent that OFETs), despite it being well known that gels can be very fluorescent and can show interesting properties to be applied in OLEDs [50]. Fluorescent gels are widely utilized as imaging agents in the field of disease diagnosis due to their three-dimensional structure, substantial water content, compatibility with biological systems, and their ability to intelligently respond to physiological triggers [51]. However, their great fluorescent properties are not reflected in a large implementation of gels in OLEDs. In spite of this, some examples in which the gel formation is used as an active layer in OLEDs have already been reported. 

**Figure 9:**
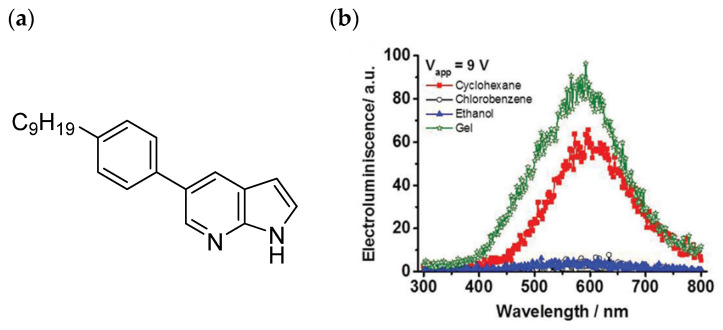
(**a**) Chemical structure of 5-(4-nonylphenyl)-7-azaindole. (**b**) Electroluminescence spectra at 9 V of OLED devices prepared using 5-(4-nonylphenyl)-7-azaindole from different solvents and with the gel fabricated from this derivative [52].

For example, Martín and co-workers [52] describe the employment of a self- organogel from 5-(4-nonylphenyl)-7-azaindole (Figure 9a), obtained via self-assembly as a new emitter in OLEDs. The gel formation is favored by hydrogen bonding. In this work, the most remarkable feature is that the gel formation changes the photophysical properties. Bearing this in mind, different OLED architectures are compared based on the single molecule and the gel formation, showing that the intramolecular interactions in the gel formed from 5-(4-nonylphenyl)-7-azaindole achieve a better efficiency in the OLED device (Figure 9b). These interesting results showed that the combination of a compound with good fluorescent properties, which can be increased by gelation, is an efficient alternative to design high-emissive OLEDs in comparison with the single ones based on organic molecules. 

This study also shows that it is not necessary to design organic molecules through hard synthetic routes with a combination of donor and acceptor groups to achieve efficient OLEDs. Easier molecules with the capacity of self-assembly and gel formation are more appropriate for the design of OLEDs from organic molecules.

**Figure 10:**
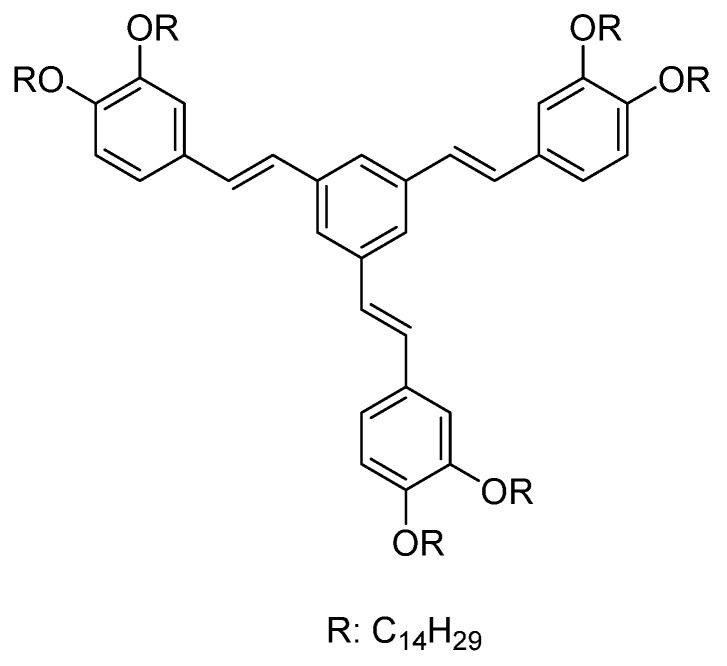
Chemical structure of the different oligo(phenylenevinylene) employed in the OLED fabrication after gel formation [53].

The electroluminescent properties of the gels of different oligo(phenylenevinylene) were also examined as emissive active layers in OLEDs employed as a host or dopant-emitters in the 4,4′-bis(N-carbazolyl)-1,1′-biphenyl host, verifying that the gel formation increases the efficiency in the OLED (Figure 10) [53].

Despite the fascinating fluorescent properties that make gels potent tools for OLEDs, their implementation in OLEDs remains surprisingly low in comparison to the utilization in other photonic and electronic devices described in this study. Several critical factors contribute to this trend. First, issues related to the durability and stability of organogels, which can degrade when exposed to environmental factors like humidity and oxygen, threaten the longevity and commercial viability of OLED devices. Secondly, the complex viscosity and processability of organogels pose manufacturing challenges, making it difficult to achieve a uniform distribution of the gel in the devices. Material compatibility is another concern, as the components of OLEDs, such as electrodes and emitting layers, must harmonize effectively with the organogel to prevent adhesion and performance issues. Although OLEDs with organogels can achieve impressive luminous efficiency, they may occasionally lag in energy efficiency compared to alternatives like LEDs. Additionally, the relatively high costs associated with the specific materials and specialized production processes required for OLEDs with organogels may limit their adoption in cost-sensitive applications. Effective thermal management is essential due to the heat generated during OLED operation and the temperature sensitivity of organogels, ensuring optimal performance and extended lifespan. Finally, recycling and disposal of OLEDs are challenging due to the presence of organic materials and precious metals, necessitating efficient methods for material separation and recovery.

It is important to note that ongoing research and development are addressing these challenges to improve the viability and applicability of OLEDs with organogels. As technology advances, solutions are likely to be found, overcoming these hurdles, and potentially opening up new opportunities in fields such as lighting and electronic displays.

One alternative in comparison with the traditional organic molecules used to form gels which can be applied in OLEDs is the employment of natural components that may form gels with potential properties to be applied in OLEDs. One interesting example are silk-fibroin-based hydrogels. Silkworms serve as a natural origin of silk, where silk primarily consists of silk fibroin and sericin in which 75% of silk is silk fibroin [54]. Silk fibroins hydrogels are highlighted by their high-water retention, self-healing ability, biocompatibility, and fluorescent properties [55]. All these characteristics make silk-fibroin-based hydrogels interesting moieties to be applied in OLEDs. In this sense, Melikov, R. et al. have already described great efficiency over 0.95 in warm white LEDs employing fibroin lenses [56]. Silk-fibroin-based hydrogels offer flexibility and they are lightweight in comparison with the rigidity of the current materials, hence they have been objects of study for researchers in the last decade [57].

## 5. Gels in Optical Waveguides

An optical waveguide is a physical structure or device designed to confine and guide light along a specific path or route. Waveguides are used in various optical and photonic applications to transmit, manipulate, and control light signals. These waveguides are typically made from materials with optical properties that allow for the internal reflection and propagation of light within the guide [58]. 

The choice of material for an optical waveguide should be based on the specific requirements of the application. A range of materials find common use in crafting optical waveguides. For instance, silicon and glass are frequently employed due to their exceptional optical transparency and minimal optical losses, rendering them proficient in light guiding. Nonetheless, in scenarios where flexibility and mechanical adaptability are paramount, these materials may not be the most suitable choices. In addition, glass waveguides, in particular, are fragile and can break or shatter upon impact. Furthermore, conventional optical waveguides lack inherent biocompatibility, necessitate intricate and precise fabrication techniques, and often involve higher production costs [59,60].

For these reasons, among these materials, gel-based optical waveguides have garnered significant attention. Their versatility arises from a combination of different properties: tunable refractive index, optical transparency, flexibility, biocompatibility, stimulus responsiveness, low cost, easy fabrication, and low propagation losses [61,62,63,64]. Additionally, the optical and mechanical characteristics of gel-based waveguides can be easily tailored by modifying factors such as polymer content, molecular weights, and crosslinking density [65,66,67].

Different molecules and/or monomers and polymers have been used in the synthesis of gel-base optical waveguides. In the case of biomedical applications, natural polymers have gained significant attention due to their inherent biocompatibility. They are well suited for use in biological and medical applications, allowing for the safe transmission of light through biological tissues and fluids. This property is invaluable in fields like biomedical imaging, where precise and non-invasive visualization is essential [68,69,70]. 

Polysaccharides such as cellulose [71], agarose [72], gelatin [73], and chitosan [74], as well as proteins like silk fibroin [75], are examples of natural polymers that have shown promise as waveguides. These waveguides have found applications in sensing, light delivery for therapy, and advanced imaging methods.

**Figure 11:**
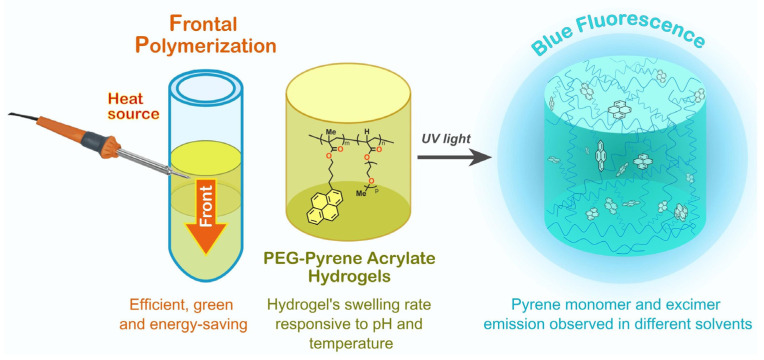
PEG hydrogels bearing pyrene groups [79]. The introduction of pyrene fluorophore gave a notable impact in the thermal properties of the hybrid hydrogels. In addition, the polarity of the solvent significantly affected the emission properties of the PEG-pyrene acrylate hydrogels. The highly efficient blue fluorescence converts these materials, promising for a wide range of applications such as sensing, photonics, or bioimaging, among others.

Nevertheless, while natural-based gels hold promise as waveguides in various applications, they also come with certain challenges and limitations focused on their poor stability or rapid degradation [76]. In response to these challenges, synthetic hydrogels (or the combination of synthetic and natural analogues) are emerging as a viable alternative. Intensive research focuses on the development of materials whose degradation releases harmless chemicals. Synthetic hydrogels offer enhanced reproducibility and mechanical strength compared to their natural counterparts [77]. Additionally, tuning the refractive index of natural-based gels to match specific optical components or requirements may be challenging due to their batch-to-batch variation [78]. Moreover, the integration of synthetic chemical structures, such as microscopic waveguides, into a macroscopic gel system proves to be a more straightforward task when dealing with synthetic gels (Figure 11) [79] or those exhibiting aggregation-induced emission phenomena, e.g., using a naphthalimide moiety [80].

Thus, considering the above information, some examples of synthetic-hybrid hydrogels are reported in this section. Some commonly used synthetic polymers for gel-based waveguides include polycaprolactone (PCL) [69], polyvinyl alcohol (PVA) [81], polyacrylamide (PAA) [82], poly(N-isopropylacrylamide) (PNIPAAm) [83], poly(L-lactic acid) (PLA) [84], and polyacrylic acid (PAAc) [85]. However, the most common is polyethylene glycol (PEG). 

**Figure 12:**
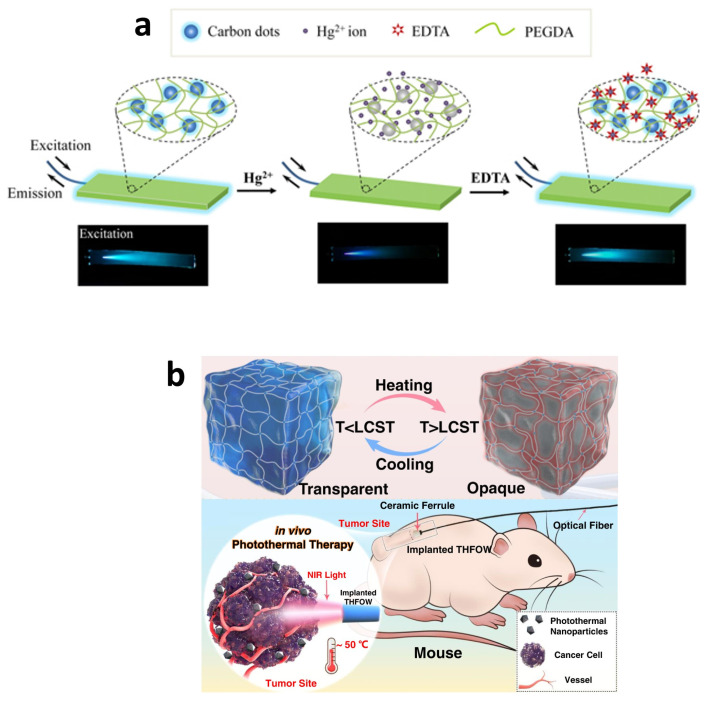
(**a**) Reversible sensing of Hg^2+^: fluorescence quenching in presence of Hg^2+^ ions and fluorescence recovery with addition of EDTA [86]. (**b**) Temperature-adaptive hydrogel fiber-based optical waveguide with in vivo applications. This method is capable of eradicating deeply seated tumor cells in mice while mitigating the risks associated with overheating, which can result in the death of healthy cells surrounding the tumor [92].

One example of these kind of hydrogels was reported by Gou et al. who prepared a waveguide PEG-based hydrogel doped with carbon dots (CDs) for the detection of Hg^2+^ in water. This hydrogel waveguide showed remarkable light-confinement properties in water, owing to the significant refractive index (RI, 1.333) contrasted with minimal light scattering losses (1.25 dB/cm). In this case, Hg^2+^ ions can penetrate the hydrogel network through diffusion and interact with CDs (Figure 12a) [86]. Other options include the incorporation of a pyrene fluorophore into PEG hydrogels, resulting in hydrogels exhibiting outstanding fluorescent properties [79], or the combination of various monomers such as PEG and PNIPAAm. These hydrogels also demonstrated pH-responsive and thermo-responsive behaviors, holding great promise for a wide range of applications, including chemical and environmental sensing [87]. Furthermore, the immobilization of diverse bioreceptors within the hydrogel waveguide opens up the potential for detecting various types of bacteria [88].

Due to its high biocompatibility and versatility, PEG is often used in biomedical applications, such as drug delivery and tissue engineering, thanks to its ability to mimic the extracellular matrix [89]. In addition, it is easily combined with acrylate functional groups, typically diacrylate molecules (PEGDA), which crosslink the PEG molecules, creating a three-dimensional network, or with natural polymers, creating a composite (e.g., with alginate, chitosan, or gelatin). PEG-based hydrogels have been harnessed for the control of analytes such as glucose [90]. Remarkably, there are potential applications of PEG-based gels for in vivo optical sensing and therapeutic applications. Specifically, a PEG-hydrogel containing cells was implanted into a mouse model afflicted with diabetes. This enabled performance of light-controlled therapy interventions aimed at enhancing glucose homeostasis. The hydrogel exhibited exceptional light-guiding capabilities, with minimal light loss (<1 dB cm^2^), while maintaining high levels of transparency and cell viability throughout the experiment [67]. In this line, photomedicine has garnered significant attention. Nevertheless, the limited depth of light penetration, a uniform refractive index, the low guiding efficiency when introduced into living biological tissues and challenges related to monitoring phototherapies have hindered their effective application in deep tissues, posing a risk to surrounding healthy tissue. To overcome these limitations, Choi et al. engineered a structure comprising a PEGDA core and an alginate clad to effectively confine light within living tissues. These core-clad hydrogel optical fibers demonstrate efficient, low-loss light guidance in vivo (<0.42 dB cm^−1^) and enable diverse optical applications, including fluorescence and photothermal effects [91]. Later, Chen et al. developed a temperature-adaptive hydrogel fiber-based optical waveguide. They can target and eliminate deeply seated tumor cells while mitigating the risk of overheating and damage to healthy tissue. Notably, the hydrogel exhibits outstanding light propagation characteristics, with an attenuation coefficient of 0.32 dB cm^−1^, and demonstrates a temperature-controlled light propagation effect (Figure 12b) [92]. In a parallel context, PLA-based bioabsorbable planar waveguides were employed in the application of photochemical tissue bonding (PTB) to treat incisions in porcine skin. This method successfully addressed full-thickness skin incisions that exceeded a depth of 1 cm [84].

Additionally, gels exhibit a high degree of versatility in terms of their shapes through a 3D-printing process [93]. For example, PEG-based waveguides were made with straightforward 3D-printing, yielding highly transparent optical waveguides. These waveguides effectively transmit light through several centimeters of porcine tissue, enabling the activation of optogenetic switches within cells and the precise regulation of cell adhesion and migration within light-responsive hydrogels (optical losses < 0.4 dB cm^−1^ in air and <0.7 dB cm^−1^ in tissues) [94]. Also, the same versatility was shown by PLA and PCL when processed using extrusion printing technology. The resulting printed waveguides exhibit optical losses around 0.02 dB cm^−1^ in air and 0.14–0.44 dB cm^−1^ in tissue. In vitro experiments demonstrate the ability of these printed waveguides to efficiently transmit light through tissue and activate photochemicals that hold relevance for biomedical applications [95].

**Figure 13:**
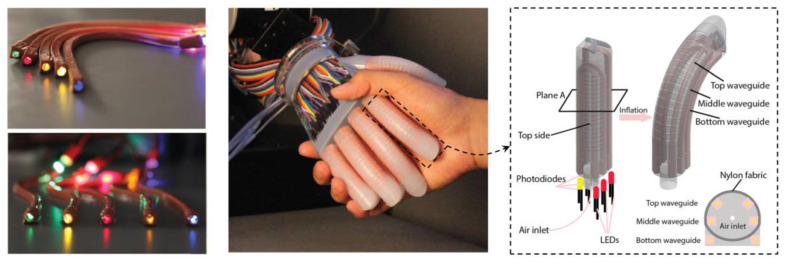
Strain-sensitive optical waveguide with a prosthetic innervated finger integrated with sensory waveguides. The incorporation of an optical waveguide into a prosthetic finger creates a soft prosthetic hand capable of providing haptic sensations. When shaking hands with this prosthetic, optical waveguides enable precise measurements of curvature, elongation, and applied force [99].

Finally, another important application for waveguide-based gels is in the field of soft robotics. Soft optical waveguide sensors have emerged as effective tools for the development of actuators and for various sensing applications, including strain, force, and bending measurements [96,97,98]. These can be effectively employed for strain sensing within a prosthetic hand, facilitating a wide array of active sensation experiments that draw inspiration from the intricate functionalities of the human hand (Figure 13) [99], or the optical waveguide deformation sensors can detect the severity of wrinkles in a thin-walled soft robot by measuring the bend angle generated in the robot [100]. Additionally, pneumatic actuators can also be produced using 3D-printing technology. In this context, Heiden et al. have devised a sustainable approach for creating these 3D-printed stretchable waveguides, enabling omnidirectional movement with response times of less than a second. These actuators integrate both proprioception (internal feedback) and exteroception (external sensing) capabilities. These soft robotic devices are equipped with dynamic real-time control systems, facilitating automated search-and-wipe routines for detecting and removing obstacles [101].

## 6. Future Perspectives

The incorporation of gels as multifunctional components within the realms of organic electronics and photonics holds immense promise for shaping the landscape of future optoelectronic technologies. As gels continue to demonstrate their prowess in enhancing charge transport, tuning energy levels, and optimizing interfacial interactions, their utilization in key areas such as organic solar cells, organic field-effect transistors (OFETs), organic light-emitting diodes (OLEDs), and optical waveguides is poised to revolutionize device performance.

In the realm of organic solar cells, gels’ ability to provide optimized morphology control and interfacial modification could pave the way for higher efficiency and stability. As new gel formulations are developed, organic solar cells may witness breakthroughs in addressing challenges related to charge extraction, exciton management, and overall device longevity.

Likewise, in the domain of OFETs, the introduction of gels as gate dielectrics or insulators could lead to enhanced charge carrier mobility, lower operation voltages, and improved device reliability. The compatibility of gels with solution processing further facilitates large-area device fabrication, bridging the gap between laboratory research and industrial scalability.

For OLEDs, the potential impact of gels is equally transformative. Gels’ role in dispersing light-emitting materials uniformly, providing protection against environmental factors, and enhancing charge injection could contribute to more efficient, vibrant, and stable OLED displays. As gels continue to evolve, the development of flexible and wearable OLEDs may become more feasible, ushering in a new era of adaptable and personalized lighting and display solutions.

Lastly, in the realm of optical waveguides, the incorporation of gels with tailored refractive indices could enable precise light confinement and manipulation, opening new avenues for efficient on-chip photonics and optical communication.

The future prospects of integrating gels into organic electronics and photonics are underscored by their versatility, adaptability, and capacity to address multifaceted challenges. As researchers delve deeper into the design of gel-based materials and their interactions within diverse device architectures, it is clear that gels will play a pivotal role in reshaping the way we harness and manipulate light, electrons, and information, forging a path towards more sustainable, efficient, and interconnected optoelectronic technologies.

## 7. Conclusions

Gels have currently become one of the elements of supramolecular chemistry with the greatest potential, and there are many articles on their possible applications. However, their applications in optoelectronics are still limited and not as well researched as in other fields.

In summary, this study has provided an in-depth insight into the fundamental role that gels play in organic electronics and photonics. Through the review of various examples and applications, we have demonstrated the versatility and promise of these materials in enhancing electronic and photonic devices based on organic materials. We have observed that gels offer an ideal platform for the fabrication of flexible and transparent devices, making them ideal candidates for wearable and portable electronic applications. Furthermore, their ability to adapt to diverse molecular structures and optical properties makes them highly valuable in organic photonics, where they can be used to modify the light-emitting properties of organic materials. We have to point out that optical waveguides are clearly the most developed field in comparison with the other three described in this work.

Our analysis has also highlighted the importance of custom gel engineering to optimize the performance of specific devices. Through careful selection of components and modulation of their physical and chemical properties, significant advancements in device efficiency and stability can be achieved.

In conclusion, gels represent an exciting and promising field in organic electronics and photonics. As we continue to research and develop new strategies for gel synthesis and application, we can anticipate significant advances in the next generation of electronic and photonic technology. This research lays the groundwork for future innovations that could have a positive impact in a wide range of applications, from flexible electronics to sensor detection and organic lighting.

Authors should discuss the results and how they can be interpreted from the perspective of previous studies and of the working hypotheses. The findings and their implications should be discussed in the broadest context possible. Future research directions may also be highlighted.

## Data Availability

Not applicable.
